# Supplementary material for: Higher- and lower-order personality traits and cluster subtypes in social anxiety disorder
Source: PLoS One. 2020 Apr 29;15(4):e0232187. doi: 10.1371/journal.pone.0232187 (PMC7190155; doi:10.1371/journal.pone.0232187)
Supplement: S1 Table — (DOCX) [file pone.0232187.s001.docx]

**S1 Table.** Comparisons of social anxiety disorder (SAD) patients and healthy controls (HC) on Revised NEO Personality Inventory facets.

|  | SAD  N=211 | HC  N=138 | t | P | *d*  vs. HC | *d*  vs. norms^1^ |
| --- | --- | --- | --- | --- | --- | --- |
|  | M (SD) | M (SD) |  |  |  |  |
| N1 Anxiety | 21.43 (6.15) | 10.04 (5.11) | 18.78 | <.001 | 2.01 | 1.36 |
| N2 Angry Hostility | 14.38 (5.09) | 8.75 (4.75) | 10.37 | <.001 | 1.14 | 0.53 |
| N3 Depression | 21.64 (5.61) | 9.78 (5.65) | 19.26 | <.001 | 2.11 | 1.02 |
| N4 Self-Consciousness | 23.45 (4.43) | 10.11 (4.55) | 27.22 | <.001 | 2.97 | 1.27 |
| N5 Impulsiveness | 16.72 (4.55) | 14.30 (5.07) | 4.65 | <.001 | 0.50 | 0.34 |
| N6 Vulnerability | 16.61 (5.48) | 7.12 (4.35) | 17.93 | <.001 | 1.92 | 0.96 |
|  |  |  |  |  |  |  |
| E1 Warmth | 15.65 (5.32) | 24.03 (4.14) | -16.48 | <.001 | -1.76 | -1.25 |
| E2 Gregariousness | 13.08 (5.88) | 21.17 (5.11) | -13.62 | <.001 | -1.47 | -0.94 |
| E3 Assertiveness | 7.83 (4.60) | 18.59 (4.82) | -20.98 | <.001 | -2.28 | -1.36 |
| E4 Activity | 14.33 (4.50) | 16.78 (4.36) | -5.02 | <.001 | -0.55 | -0.64 |
| E5 Excitement seeking | 14.25 (5.65) | 18.10 (4.63) | -6.95 | <.001 | -0.74 | -0.03 |
| E6 Positive Emotions | 15.36 (6.53) | 24.81 (5.41) | -14.69 | <.001 | -1.57 | -0.97 |
|  |  |  |  |  |  |  |
| O1 Fantasy | 18.13 (5.71) | 19.04 (5.99) | -1.43 | *.154* | -0.15 | 0.32 |
| O2 Aesthetics | 14.81 (7.24) | 16.43 (7.38) | -2.03 | *.043* | -0.22 | -0.13 |
| O3 Feelings | 20.38 (5.62) | 21.78 (4.83) | -2.47 | *.014* | -0.27 | 0.02 |
| O4 Actions | 13.34 (5.20) | 18.57 (4.96) | -9.34 | <.001 | -1.03 | -0.47 |
| O5 Ideas | 17.51 (6.53) | 20.76 (5.79) | -4.75 | <.001 | -0.56 | 0.25 |
| O6 Values | 23.21 (3.36) | 24.68 (4.24) | -3.43 | <.001 | -0.38 | 0.61 |
|  |  |  |  |  |  |  |
| C1 Competence | 18.41 (4.50) | 24.89 (4.03) | -13.69 | <.001 | -1.52 | -0.74 |
| C2 Order | 17.40 (4.79) | 18.30 (4.27) | -1.78 | .*075* | -0.17 | -0.37 |
| C3 Dutifulness | 23.19 (4.41) | 25.12 (4.17) | -4.07 | <.001 | -0.45 | -0.33 |
| C4 Achievement striving | 15.73 (4.82) | 17.07 (4.81) | -2.54 | .*012* | -0.26 | -0.17 |
| C5 Self-discipline | 15.36 (6.25) | 21.37 (5.51) | -9.43 | <.001 | -1.02 | -0.91 |
| C6 Deliberation | 19.55 (5.26) | 19.52 (5.31) | .06 | .*955* | 0.01 | 0.07 |
|  |  |  |  |  |  |  |
| A1 Trust | 17.96 (5.74) | 23.83 (4.79) | -10.33 | <.001 | -1.11 | -0.66 |
| A2 Straightforwardness | 22.38 (4.61) | 19.67 (5.07) | 5.16 | <.001 | 0.56 | 0.19 |
| A3 Altruism | 22.99 (3.88) | 25.79 (3.93) | -6.56 | <.001 | -0.72 | -0.22 |
| A4 Compliance | 21.25 (4.89) | 19.62 (7.23) | 2.50 | .*013* | 0.26 | 0.51 |
| A5 Modesty | 23.51 (4.43) | 19.65 (4.88) | 7.64 | <.001 | 0.83 | 0.44 |
| A6 Tender-mindedness | 23.22 (4.68) | 23.27 (4.39) | -.09 | .*928* | -0.01 | 0.005 |

NEO-PI-R = Revised NEO Personality Inventory; *d* = effect size according to Cohen’s *d*;

Holm adjusted $=.00625-.0017;$ ^1^SAD in comparison to Swedish normative data [67].
