# Supplementary material for: Higher- and lower-order personality traits and cluster subtypes in social anxiety disorder
Source: PLoS One. 2020 Apr 29;15(4):e0232187. doi: 10.1371/journal.pone.0232187 (PMC7190155; doi:10.1371/journal.pone.0232187)
Supplement: S2 Table — (DOCX) [file pone.0232187.s002.docx]

**S2 Table***.* Correlations between Karolinska Scales of Personality items and the Revised NEO Personality Inventory dimensions in the social anxiety disorder group.

|  | Neuroticism | Extraversion | Openness | Agreeableness | Conscientiousness |
| --- | --- | --- | --- | --- | --- |
| Psychic Anxiety | .708** | -.399** | -.087 | .058 | -.168* |
| Somatic Anxiety | .563** | -.081 | .106 | .000 | -.127 |
| Psychasthenia | .477** | -.330** | -.095 | -.008 | -.318** |
| Inhibition of Aggression | .417** | -.375** | -.119 | .316** | -.170* |
| Detachment | .103 | -.578** | -.361** | -.205** | -.138 |
| Muscular Tension | .435** | -.121 | -.153* | -.066 | -.053 |
| Irritability | .502** | -.363** | -.126 | -.431** | -.208** |
| Suspicion | .368** | -.070 | .002 | -.336** | -.029 |
| Socialization | -.398** | .023 | -.098 | .261** | .162* |
| Guilt | .547** | -.108 | .017 | .073 | -.116 |
| Monotony Avoidance | -.129 | .585** | .387** | -.148 | .141 |
| Impulsivity | -.100 | .432** | .119 | -.189* | -.344** |
| Social Desirability | -.222** | .044 | .017 | .461** | .123 |
| Verbal Aggression | .040 | .247** | .081 | -.560** | -.071 |
| Indirect Aggression | .393** | .048 | -.048 | -.234** | -.109 |

** Correlations significant at the $p<0.01$ level (2-tailed)

* Correlations significant at the $p<0.05$ level (2-tailed)
