# Supplementary material for: Higher- and lower-order personality traits and cluster subtypes in social anxiety disorder
Source: PLoS One. 2020 Apr 29;15(4):e0232187. doi: 10.1371/journal.pone.0232187 (PMC7190155; doi:10.1371/journal.pone.0232187)
Supplement: S3 Table — (DOCX) [file pone.0232187.s003.docx]

**S3 Table.** Mean values (SD) and ANOVA results on the Revised NEO Personality Inventory facets in three clusters of social anxiety disorder (SAD) patients in comparison to healthy controls (HC).

|  | (n=138)  HC | 1 (n=69)  Prototypical | 2 (n=62)  Introvert-  Conscientious | 3 (n=80)  Instable-  Open | F  (3, 348) | $P$ | Post-hoc |
| --- | --- | --- | --- | --- | --- | --- | --- |
| N1 Anxiety  *d* vs. norms^1^  description^1^ | 10.04 (5.11) | 24.58 (4.51)  2.17  Very high | 17.23 (6.44)  0.64  Moderately high | 21.98 (5.30)  1.56  Very high | 149.82 | <.001 | HC<2<3<1 |
| N2 Hostility  *d* vs. norms^1^  description^1^ | 8.75 (4.75) | 16.54 (4.67)  1.02  High | 11.15 (4.13)  0.15  Average | 15.03 (4.93)  0.68  Moderately high | 55.63 | <.001 | HC<2<(3=1) |
| N3 Depression  *d* vs. norms^1^  description^1^ | 9.78 (5.65) | 24.91 (4.09)  2.35  Very high | 17.65 (5.05)  0.78  Moderately high | 21.91 (5.25)  1.57  Very high | 167.57 | <.001 | HC<2<3<1 |
| N4 Self-consciousness  *d* vs. norms^1^  description^1^ | 10.11 (4.55) | 25.49 (3.33)  2.93  Very high | 21.92 (4.09)  1.91  Very high | 22.89 (4.89)  1.96  Very high | 270.91 | <.001 | HC<(2=3)<1 |
| N5 Impulsiveness  *d* vs. norms^1^  description^1^ | 14.30 (5.07) | 17.30 (4.19)  0.49  Slightly high | 13.74 (4.01)  -0.35  Slightly low | 18.53 (4.10)  0.79  Moderately high | 21.68 | <.001 | (HC=2)<(1=3) |
| N6 Vulnerability  *d* vs. norms^1^  description^1^ | 7.12 (4.35) | 20.10 (4.40)  2.09  Very high | 13.06 (4.52)  0.48  Slightly high | 16.34 (5.17)  1.13  Very high | 144.37 | <.001 | HC<2<3<1 |
|  |  |  |  |  |  |  |  |
| E1 Warmth  *d* vs. norms^1^  description^1^ | 24.03 (4.14) | 13.62 (4.50)  -1.83  Very low | 13.81 (4.68)  -1.76  Very low | 18.84 (4.93)  -0.62  Moderately low | 117.94 | <.001 | HC>3>(2=1) |
| E2 Gregariousness  *d* vs. norms^1^  description^1^ | 21.17 (5.11) | 10.96 (5.33)  -1.38  Very low | 10.81 (5.34)  -1.40  Very low | 16.66 (4.95)  -0.36  Slightly low | 89.22 | <.001 | HC>3>(2=1) |
| E3 Assertiveness  *d* vs. norms^1^  description^1^ | 18.59 (4.82) | 5.30 (3.25)  -2.08  Very low | 7.95 (4.44)  -0.84  Low | 9.93 (4.67)  -0.93  Low | 176.18 | <.001 | HC>(3=2)>1 |
| E4 Activity  *d* vs. norms^1^  description^1^ | 16.78 (4.36) | 12.51 (3.67)  -1.18  Very low | 13.18 (3.84)  -0.99  Low | 16.79 (4.57)  -0.07  Average | 24.50 | <.001 | (HC=3)>(2=1) |
| E5 Excitement-seeking  *d* vs. norms^1^  description^1^ | 18.10 (4.63) | 11.78 (4.93)  -0.47  Slightly low | 11.94 (4.98)  -0.47  Slightly low | 18.18 (4.42)  0.76  Moderately high | 48.43 | <.001 | (HC=3)>(2=1) |
| E6 Positive Emotions  *d* vs. norms^1^ | 24.81 (5.41) | 11.57 (5.54)  -1.74 | 13.56 (6.02)  -1.32 | 20.01 (4.68)  -0.23 | 118.86 | <.001 | HC>3>(2=1) |
| description^1^ |  | Very low | Very low | Slightly low |  |  |  |
|  |  |  |  |  |  |  |  |
| O1 Fantasy  *d* vs. norms^1^  description^1^ | 19.04 (5.99) | 16.46 (4.94)  0.47  Slightly high | 15.81 (5.39)  -0.11  Average | 21.38 (5.10)  0.97  High | 15.88 | <.001 | 3>HC>(2=1) |
| O2 Aesthetics  *d* vs. norms^1^  description^1^ | 16.43 (7.38) | 12.07 (6.98)  -0.53  Moderately low | 12.97 (6.78)  -0.04  Average | 18.60 (6.18)  0.45  Slightly high | 14.52 | <.001 | (HC=3)>(2=1) |
| O3 Feelings  *d* vs. norms^1^  description^1^ | 21.78 (4.83) | 18.32 (5.31)  -0.39  Slightly low | 18.15 (5.75)  -0.41  Slightly low | 23.89 (3.73)  0.85  High | 24.43 | <.001 | 3>HC>(2=1) |
| O4 Actions  *d* vs. norms^1^  description^1^ | 18.57 (4.96) | 11.12 (4.70)  -0.96  Low | 12.50 (4.72)  -0.67  Moderately low | 15.91 (4.91)  0.04  Average | 45.10 | <.001 | HC>3>(2=1) |
| O5 Ideas  *d* vs. norms^1^  description^1^ | 20.76 (5.79) | 14.07 (6.07)  -0.33  Slightly low | 17.52 (6.16)  0.26  Slightly high | 20.48 (5.76)  0.79  Moderately high | 22.10 | <.001 | (HC=3)>2>1 |
| O6 Values  *d* vs. norms^1^  description^1^ | 24.68 (4.24) | 22.33 (3.52)  0.34  Slightly high | 22.92 (3.54)  0.51  Moderately high | 24.20 (2.83)  0.96  High | 7.75 | <.001 | (HC=3)>(2=1) |
|  |  |  |  |  |  |  |  |
| A1 Trust  *d* vs. norms^1^  description^1^ | 23.83 (4.79) | 16.20(6.08)  -0.97  Low | 18.58 (5.79)  -0.53  Moderately low | 19.00 (5.07)  -0.49  Slightly low | 37.70 | <.001 | HC>3>1 |
| A2 Straightforwardness  *d* vs. norms^1^  description^1^ | 19.67 (5.07) | 23.17 (4.36)  0.36  Slightly high | 23.66 (4.58)  0.46  Slightly high | 20.70 (4.39)  -0.17  Average | 14.87 | <.001 | (1=2)>(HC=3) |
| A3 Altruism  *d* vs. norms^1^  description^1^ | 25.79 (3.93) | 22.04 (4.48)  -0.43  Slightly low | 22.82 (3.16)  -0.29  Slightly low | 23.94 (3.64)  0.04  Average | 17.66 | <.001 | HC>3>1 |
| A4 Compliance  *d* vs. norms^1^  description^1^ | 19.62 (7.23) | 22.00 (4.21)  0.72  Moderately high | 21.66 (4.57)  0.61  Moderately high | 20.28 (5.54)  0.28  Slightly high | 3.29 | .021 | 1>HC |
| A5 Modesty  *d* vs. norms^1^  description^1^ | 19.65 (4.88) | 25.07 (4.00)  0.82  High | 23.45 (4.25)  0.43  Slightly high | 22.21 (4.54)  0.15  Average | 25.11 | <.001 | (1=2)>3>HC |
| A6 Tender-mindedness  *d* vs. norms^1^  description^1^ | 23.27 (4.39) | 23.10 (4.70)  -0.04  Average | 22.16 (4.52)  -0.27  Slightly low | 24.15 (4.70)  0.20  Slightly high | 2.26 | .081 | HC=1=2=3 |
| C1 Competence  *d* vs. norms^1^  description^1^ | 24.89 (4.03) | 14.90 (3.67)  -1.77  Very low | 21.40 (2.90)  -0.03  Average | 19.13 (4.18)  -0.61  Moderately low | 112.99 | <.001 | HC>2>3>1 |
| C2 Order  *d* vs. norms^1^  description^1^ | 18.30 (4.27) | 16.38 (4.85)  -0.59  Moderately low | 20.44 (3.70)  0.33  Slightly high | 15.93 (4.47)  -0.72  Moderately low | 15.66 | <.001 | 2>HC>(3=1) |
| C3 Dutifulness  *d* vs. norms^1^  description^1^ | 25.12 (4.17) | 21.61 (4.13)  -0.73  Moderately low | 25.60 (3.09)  0.27  Slightly high | 22.69 (4.76)  -0.43  Slightly low | 16.74 | <.001 | (HC=2)>(3=1) |
| C4 Achievement-striving  *d* vs. norms^1^  description^1^ | 17.07 (4.81) | 13.58 (4.22)  -0.68  Moderately low | 17.16 (4.50)  0.15  Average | 16.49 (4.96)  -0.02  Average | 9.75 | <.001 | (HC=2=3)>1 |
| C5 Self-discipline  *d* vs. norms^1^  description^1^ | 21.37 (5.51) | 11.59 (5.43)  -1.72  Very low | 19.02 (5.18)  -0.29  Slightly low | 15.79 (5.88)  -0.87  Low | 52.72 | <.001 | HC>2>3>1 |
| C6 Deliberation  *d* vs. norms^1^  description^1^ | 19.52 (5.31) | 18.70 (5.13)  -0.10  Average | 22.87 (4.78)  -0.75  Moderately low | 17.73 (4.55)  -0.30  Slightly low | 13.26 | <.001 | 2>HC |

*d* = between-group effect size according to Cohen’s *d*; ^1^SAD in comparison to Swedish norm data [67].
