# Supplementary material for: Higher- and lower-order personality traits and cluster subtypes in social anxiety disorder
Source: PLoS One. 2020 Apr 29;15(4):e0232187. doi: 10.1371/journal.pone.0232187 (PMC7190155; doi:10.1371/journal.pone.0232187)
Supplement: S4 Table — (DOCX) [file pone.0232187.s004.docx]

**S4 Table.** Mean values (SD) and ANOVA results on the Karolinska Scales of Personality variables in the three clusters of social anxiety disorder (SAD) patients in comparison to healthy controls (HC).

|  | (n=124)  HC | 1 (n=60)  Prototypical | 2 (n=41)  Introvert- Conscientious | 3 (n=63)  Instable-  Open | F  (3, 162) | $P$ | Post-hoc |
| --- | --- | --- | --- | --- | --- | --- | --- |
| Psychic Anxiety  *d* vs. norms^1^  description^1^ | 16.79 (4.93) | 32.12 (3.41)  2.53  Very high | 28.49 (5.34)  1.40  Very high | 28.84 (4.74)  1.56  Very high | 193.27 | <.001 | HC<(2=3)<1 |
| Somatic Anxiety  *d* vs. norms^1^  description^1^ | 14.07 (3.81) | 25.43 (4.94)  1.74  Very high | 21.10 (4.67)  0.88  High | 24.16 (5.41)  1.41  Very high | 115.76 | <.001 | HC<2<(1=3) |
| Psychasthenia  *d* vs. norms^1^  description^1^ | 18.48 (4.33) | 28.55 (3.88)  1.96  Very high | 24.76 (3.94)  0.98  High | 25.19 (4.49)  1.01  High | 90.04 | <.001 | HC<(2=3)<1 |
| Inhibition of Aggression  *d* vs. norms^1^  description^1^ | 21.74 (4.09) | 31.32 (4.49)  1.75  Very high | 28.63 (4.49)  1.16  Very high | 28.40 (5.49)  0.99  High | 73.28 | <.001 | HC<(2=3)<1 |
| Detachment  *d* vs. norms^1^  description^1^ | 18.29 (4.04) | 26.87 (3.92)  1.39  Very high | 26.71 (4.47)  1.25  Very high | 22.38 (5.14)  0.20  Slightly high | 71.35 | <.001 | HC<3<(1=2) |
| Muscular Tension  *d* vs. norms^1^  description^1^ | 14.23 (4.65) | 23.70 (5.58)  1.50  Very high | 18.76 (4.82)  0.59  Moderately high | 20.86 (5.17)  0.99  High | 56.38 | <.001 | HC<(2=3)<1 |
| Irritability  *d* vs. norms^1^  description^1^ | 9.68 (2.28) | 13.73 (1.62)  1.09  High | 11.76 (2.61)  0.11  Average | 12.06 (2.39)  0.25  Slightly high | 48.29 | <.001 | HC<(2=3)<1 |
| Suspicion  *d* vs. norms^1^  description^1^ | 8.00 (2.30) | 11.88 (2.54)  0.98  High | 10.20 (2.67)  0.28  Slightly high | 11.48 (2.75)  0.77  Moderately high | 44.81 | <.001 | HC<(1=3) |
| Socialization  *d* vs. norms^1^  description^1^ | 68.83 (9.02) | 56.63 (8.51)  -1.23  Very low | 63.41 (8.42)  -0.42  Slightly low | 57.25 (9.44)  -1.09  Low | 36.53 | <.001 | HC>2>(1=3) |
| Guilt  *d* vs. norms^1^  description^1^ | 10.69 (2.03) | 13.35 (2.18)  0.75  Moderately high | 11.56 (1.64)  0.05  Average | 12.71 (2.34)  0.45  Slightly high | 27.06 | <.001 | (HC=2)<(1=3) |
| Monotony Avoidance  *d* vs. norms^1^  description^1^ | 25.83 (4.93) | 19.20 (4.59)  -0.80  Low | 20.12 (4.02)  -0.64  Moderately low | 25.37 (5.47)  0.47  Slightly high | 34.73 | <.001 | (HC=3)>(1=2) |
| Impulsivity  *d* vs. norms^1^  description^1^ | 23.65 (4.53) | 20.30 (4.15)  -0.63  Moderately low | 18.05 (4.63)  -1.11  Very low | 22.35 (4.07)  -0.14  Average | 20.15 | <.001 | (HC=3)>2 |
| Social Desirability  *d* vs. norms^1^  description^1^ | 28.57 (3.70) | 26.17 (3.82)  NA | 28.41 (3.11)  NA | 26.70 (3.97)  NA | 7.72 | <.001 | HC>(1=3) |
| Verbal Aggression  *d* vs. norms^1^  description^1^ | 11.56 (2.76) | 10.15 (2.94)  -0.87  Low | 9.59 (2.53)  -1.15  Very low | 11.13 (2.97)  -0.53  Moderately low | 6.84 | <.001 | HC=1,2; 2=3 |
| Indirect Aggression  *d* vs. norms^1^  description^1^ | 11.52 (2.76) | 12.80 (3.14)  0.37  Slightly high | 10.80 (2.22)  -0.36  Slightly low | 11.95 (2.93)  0.08  Average | 4.71 | .003 | 1>2 |

^1^SAD in comparison to Swedish norm data, Bergman et al. [68].
